# Editorial: Research topic for frontiers in cardiovascular medicine: non-invasive sensing and imaging techniques for cardiometabolic diseases

**DOI:** 10.3389/fcvm.2023.1178101

**Published:** 2023-05-15

**Authors:** A. Karlas, M. Bariotakis, M. Kallmayer, L. Hadjileontiadis, M. Wildgruber

**Affiliations:** ^1^ Institute of Biological and Medical Imaging, Helmholtz Zentrum München, Neuherberg, Germany; ^2^ Chair of Biological Imaging at the Central Institute for Translational Cancer Research (TranslaTUM), Technical University of Munich, Munich, Germany; ^3^ Department for Vascular and Endovascular Surgery, Klinikum rechts der Isar, Technical University of Munich, Munich, Germany; ^4^ DZHK (German Centre for Cardiovascular Research), Partner Site Munich Heart Alliance, Munich, Germany; ^5^ Department of Biomedical Engineering, Khalifa University of Science and Technology, Abu Dhabi, United Arab Emirates; ^6^ Healthcare Innovation Center, Khalifa University of Science and Technology, Abu Dhabi, United Arab Emirates; ^7^ Department of Electrical and Computer Engineering, Aristotle University of Thessaloniki, Thessaloniki, Greece; ^8^ Department of Radiology, University Hospital, Ludwig Maximilian University of Munich, Munich, Germany

**Keywords:** sensing, imaging, diagnosis, clinical management, cardiometabolic diseases

**Editorial on the Research Topic** Research topic for frontiers in cardiovascular medicine: non-invasive sensing and imaging techniques for cardiometabolic diseases

Management of cardiometabolic diseases consists one of the biggest challenges in medicine. The heterogeneity of such diseases renders their clinical management complex and the development of tools for assessment of specific treatment effects necessary. Non-invasive sensing or imaging techniques play an important role in the in evaluating novel treatment options such as anti-inflammatory regimens or cell-based therapies. In fact, by directly assessing the affected organs and tissues such techniques frequently offer better specificity than routine blood analyses. For example, the diagnosis of several heart conditions is based mainly on the interpretation of electrocardiographic or echocardiographic readouts. Also, the detection and stratification of non-alcoholic fatty liver disease relies on direct imaging of the liver.

**Figure 1:**
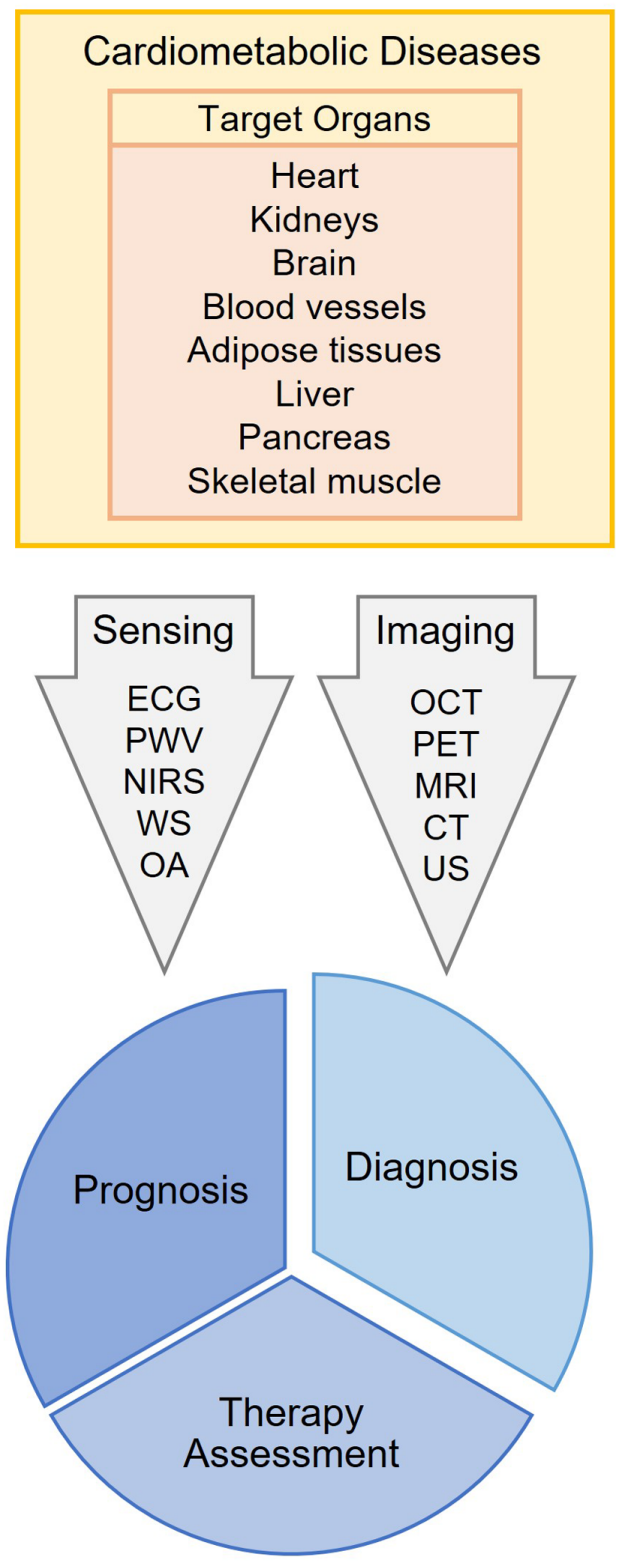
General idea of the current research topic. ECG, Electrocardiography; PWV, Pulse Wave Analysis; NIRS, Near-Infrared Spectroscopy; WS, Wearable Sensors; OA, Optoacoustics; OCT, Optical Coherence Tomography; PET, Positron Emission Tomography; MRI, Magnetic Resonance Imaging; CT, Computed Tomography; US, Ultrasound.

The Research Topic “Non-invasive Sensing and Imaging Techniques for Cardiometabolic Diseases”, with five original articles and two reviews, covered several clinical and translational aspects of the techniques already used or show potential to be used in the management of cardiometabolic diseases (Figure 1).

The study of Wang et al. explores the connection between preinfraction angina (PIA) and the morphological characteristics of the vulnerable coronary plaque in patients with ST-segment elevation myocardial infraction (STEMI). Optical coherence tomography (OCT), an intravascular high-resolution (10–20 um) imaging technique, revealed a lower rate of plaque rupture and increased lipid content in vulnerable plaques in patients with STEMI and PIA, compared to patients with STEMI but no PIA. The study not only provides insights into the pathophysiology of STEMI, by implying different pathophysiologic mechanisms between the two groups, but has also clinical implications by demonstrating that the absence of PIA is an independent predictor of plaque rupture in STEMI patients.

In the work of Zhao et al. is addressed the echocardiographic differentiation between hypertrophic cardiomyopathy (HCM) and hypertensive left ventricular hypertrophy (H-LVH): two conditions with different pathophysiology, prognosis and treatment. To this end, the ultrasound-based non-invasive myocardial work index (NMWI)—an index derived from several pressure-strain-loops-based parameters, such as the global myocardial work index (GWI) and global constructive work (GCW)—was compared to other conventional echocardiographic readouts, such as the inter-ventricular septum thickness (IVST) and left ventricular posterior wall thickness (LVPWT), in 40 patients with HCM and 35 patients with H-LVH. It was shown that NMWI is characterized by a significant difference between the two groups, offering possibly novel insights into the pathophysiology of both diseases. Moreover, even if both IVST/LVPWT and GCW were proven to be predictors for discriminating the two forms of hypertrophy, combining these two parameters provided higher predictive accuracy than using each parameter on its own. This finding further highlights the clinical implications of the study in distinguishing between HCM and H-LVH based purely on echocardiography-extracted measurements.

Xie et al. investigate the relationship between the fragmented QRS (f-QRS) complex of the surface ECG signals with the functional status of the myocardium in patients with previously diagnosed Danon disease (DD): a genetic metabolic disorder associated with cardiomyopathy. *N* = 15 patients in total underwent 12-lead ECG for the assessment of the f-QRS complex and heart magnetic resonance imaging (CMR) to evaluate the cardiac functional status *via* parameters, such as the left ventricular ejection fraction (LVEF) and the late gadolinium enhancement (LGE), a characteristic of irreversible myocardial fibrosis or scarring and, thus, loss of functionality. The results showed that the f-QRS were commonly seen in patients with DD and associated with a higher level of myocardial fibrosis, as described by LGE > 35% in CMR. Finally, the f-QRS showed in general a positive correlation with myocardial fibrosis, as described by LGE, and a negative correlation with myocardial functionality, as described by LVEF.

In another interesting study, Lohr et al. aim at characterizing the myocardial microstructure and function in a small animal model of obesity-associated heart dysfunction. Male C57BL/6N mice were either fed with a high-fat diet (HFD, *N* = 12) or normal control diet (NCD, *N* = 10) for 15 weeks. Mouse hearts were assessed both functionally (*in vivo* ultra-high frequency echocardiography, UHFE) and microanatomically by means of ex vivo diffusion tensor magnetic resonance imaging (DT-MRI), a technique that offers a resolution of 100 um. The latter allowed for a depth analysis of myocardial deformation, diffusion metrics and fiber tract geometry of the myocardium. DT-MRI revealed distorted myocardial diffusion properties in the HFD group but no changes in the three-dimensional (3D) arrangement of the myocardial fibers of the left ventricle (LV). Furthermore, obesity was correlated with reduced longitudinal strain and mean diffusivity in the basal region of the LV, compared to the control group. The study highlights the high potential of DT-MRI as a novel tool in the assessment and diagnostics of heart dysfunction in obesity.

In the original research paper of Chamtouri et al. it is shown that high severity of severe acute respiratory syndrome coronavirus 2 (SARS-CoV-2), as reflected in computed tomography (CT), is associated with higher possibility to develop subclinical heart damage, as described in echocardiographic measurements. More specifically, *N* = 111 subjects recovered from COVID-19 were included in the study. All subjects underwent a chest CT scan and, based on this, were categorized in subjects with mild CT scan lesions (<50%) and with severe CT scan lesions (≥50%). Finally, after three months all participants underwent blood analysis to estimate the levels of high-sensitivity cardiac troponin (hs-cTn) and transthoracic echocardiography (TTE) to measure the global longitudinal strain (GLS) of the myocardium: a parameter reflecting the function of the longitudinal fibers in the subendocardium. The outcomes showed that subjects with severe CT scan lesions are more prone to develop subclinical myocardial damage, possibly leading to the useful clinical recommendation that patients recovering from COVID-19 could undergo a TTE examination to check for silent cardiac lesions.

Furthermore, two review papers are published in this research topic. In the study of Chalet et al. the authors provide an overview of the methods used in imaging of ischemic penumbra, the severely hypoperfused but not yet infarcted brain region after an acute ischemic stroke (AIS), while exploring future directions. Imaging the penumbra is critical for guiding the acute actions which might allow for achieving reperfusion and improving the functional outcome. Apart from ^15^O-positron emission tomography (^15^O-PET), the current gold standard method, the use of multiparametric magnetic resonance imaging (MRI) and computed tomography (CT) are also presented and thoroughly discussed. The evolution of penumbra imaging is described over time and in correlation with the main therapeutic strategies, i.e., thrombolysis and thrombectomy. While highlighting the role of imaging within the clinical emergency settings, the authors provide insights into promising clinical imaging biomarkers/methods and potential research targets, such as the MR-based CMRO_2_ (cerebral metabolic rate of oxygen) and the MR vascular fingerprinting method.

In the second review paper, Kampaktsis et al. provide a comprehensive overview of applications of artificial intelligence (AI) in the management of atherosclerosis, a condition strongly related to cardiometabolic disease. First, the key concepts of AI applications are described from a clinical point of view. Second, a detailed overview of AI applications is given for four main categories of atherosclerotic cardiovascular disease (ASCVD): coronary artery disease (CAD), peripheral arterial disease (PAD), abdominal aortic aneurysm (AAA), and carotid artery disease. For each type of ASCVD, Kampaktsis et al. focus on the main imaging modalities employed in clinical practice as well as the key aim of the applied AI techniques, i.e., disease detection, phenotyping, prediction of outcome and assistance of clinical decision process. Finally, advantages and disadvantages of current AI approaches are discussed and future perspectives are provided.

To summarize, the current Research Topic for Frontiers in Cardiovascular Medicine: Non-invasive Sensing and Imaging Techniques for Cardiometabolic Diseases provides the reader with a comprehensive overview of sensing and imaging applications in preclinical and translational research and clinical management of cardiometabolic diseases. Non-invasive imaging approaches will play a major role in driving the research, optimising diagnosis and personalising the therapy of the patient with cardiometabolic disease. Clearly, imaging may provide a high-resolution assessment, both in space and in time, of cardiometabolic alterations and the effects of novel targeted therapies. Therefore, further development, evaluation and clinical translation of such techniques is important to foster advances in the diagnosis and treatment of cardiovascular disease.

